# Safety and Effectiveness of Uterine Gauze Packing for Refractory Postpartum Haemorrhage: A Systematic Review and Meta‐Analysis

**DOI:** 10.1111/1471-0528.70091

**Published:** 2025-11-21

**Authors:** Maureen Makama, Alicia Ferguson, Mairead Connolly, Sarah Boudova, Samia Aziz, Lorena Romero, Joshua P. Vogel

**Affiliations:** ^1^ Women's, Children's and Adolescents' Health Program Burnet Institute Melbourne Australia; ^2^ School of Public Health and Preventive Medicine Monash University Melbourne Australia; ^3^ Department of Obstetrics and Gynecology, Division of Maternal, Fetal Medicine Thomas Jefferson University Philadelphia Pennsylvania USA; ^4^ The Ian Potter Library Alfred Health Melbourne Australia

**Keywords:** haemostatic agents, impregnated gauze, plain gauze, postpartum haemorrhage, uterine packing

## Abstract

**Background:**

The role of uterine gauze packing in treating refractory postpartum haemorrhage (PPH) is uncertain given limited evidence of benefit and possible harms.

**Objectives:**

To evaluate the safety and effectiveness of uterine packing using plain gauze or gauze impregnated with haemostatic agent(s) for treating refractory PPH.

**Search Strategy:**

We searched MEDLINE, Embase, Emcare, Web of Science, CINAHL and CENTRAL from inception through March 2025.

**Selection Criteria:**

Randomised controlled trials (RCTs) and non‐randomised controlled studies of intervention (NRSIs) evaluating uterine packing using plain gauze or gauze impregnated with haemostatic agents, compared with usual care or other interventions, for women with refractory PPH.

**Data Collection and Analysis:**

We included one RCT (204 women) and six NRSIs (814 women). We presented findings from the RCT in a forest plot and conducted random‐effects meta‐analyses for NRSIs.

**Main Results:**

Three comparisons had data: plain gauze versus balloon tamponade; gauze impregnated with haemostatic agents versus balloon tamponade; and plain gauze versus uterine artery ligation/embolisation. From the RCT, uterine packing with plain gauze may not reduce the need for additional surgical/radiological intervention (relative risk (RR) 1.29, 95% CI 0.50–3.32) or blood transfusion (RR 0.97, 95% CI 0.67–1.41; *low certainty evidence*). Moderate‐certainty evidence indicates that for women with refractory PPH, uterine packing with plain gauze probably increases postpartum anaemia (< 11 g/dL) (RR 1.27, 95% CI 1.07–1.51) and maternal fever > 38°C (RR 2.40, 95% CI 1.21–4.76), compared to balloon tamponade. From NRSIs, evidence is very uncertain about the effects of uterine packing with plain gauze or gauze impregnated with haemostatic agents on all outcomes (*very low certainty evidence*).

**Conclusion:**

There are no clear benefits of uterine packing with plain gauze or gauze impregnated with haemostatic agents for treating refractory PPH, and the risk of postpartum anaemia and maternal fever is probably increased compared to standard care. Prospective well‐controlled trials are needed to evaluate the effects of uterine packing and its role in treating refractory PPH.

## 1 Introduction

Postpartum haemorrhage (PPH) remains the leading direct cause of maternal death, accounting for an estimated 15% of maternal mortality globally [1, 2]. Despite being largely preventable, PPH resulted in more than 570 000 maternal deaths between 2009 and 2020, with women in low‐ and middle‐income countries (LMICs) disproportionately affected [2, 3]. Early detection and timely evidence‐based management are crucial to reducing maternal mortality and morbidity from PPH [4, 5].

Currently, the World Health Organization (WHO) recommends the prophylactic administration of uterotonics (10 IU oxytocin given IM or IV) during the third stage of labour to prevent PPH [4]. If PPH occurs, administration of uterotonics, uterine massage, tranexamic acid and fluid resuscitation are recommended as first‐line treatments [4, 6, 7]. Although most patients respond to these first‐line treatments, bleeding persists in 10%–20% of cases (referred to as refractory PPH) requiring additional intervention [8]. For refractory PPH due to uterine atony, the recommended second‐line treatments include, intrauterine balloon tamponade, uterine artery embolisation, bimanual uterine compression and external aortic compression [4, 6]. When these measures fail to stop bleeding, surgical intervention starting with conservative approaches (compression sutures, uterine, utero‐ovarian and hypogastric vessel ligation) before hysterectomy is recommended. Uterine packing is a procedure where gauze or specialised packing material is inserted into the uterus to put direct pressure on the placental bed to control severe PPH [9]. In the 2012 WHO recommendation, uterine packing was not recommended for the treatment of PPH due to uterine atony after vaginal birth [4]. This was a weak recommendation due to a lack of quality evidence of its benefit and its potential to cause harm [4].

To reinvigorate efforts to address PPH, in 2023 WHO convened a Global Summit on PPH which resulted in the establishment of a roadmap to combat PPH between 2023 and 2030 [3]. A central component of this roadmap was the development of consolidated PPH guidelines. This harmonised and innovative approach aims to address inconsistencies across guidelines, and duplication of efforts to ensure that recommendations are comprehensive and reliable. To inform the consolidated guidelines, this review aimed to synthesise the available evidence on the use of uterine packing with plain gauze or gauze impregnated with haemostatic agents for treating refractory PPH.

## 2 Methods

The review protocol was prospectively registered in the Prospective Register of Systematic Reviews (PROSPERO) (CRD420251017688), and findings reported following the PRISMA guidelines and Cochrane Handbook for Systematic Reviews [10].

### 2.1 Eligibility Criteria, Information Sources and Search Strategy

Randomised controlled trials (RCTs) and non‐randomised controlled studies of intervention (NRSIs) of uterine packing using plain gauze or gauze impregnated with haemostatic agents for treating refractory PPH, irrespective of mode of delivery were eligible. Only studies with a comparison group including usual care, other interventions for refractory PPH (e.g., uterine balloon tamponade) or no intervention, were included. Eligible NRSIs included observational studies such as cohort, case–control and cross‐sectional studies. Case series, case reports, reviews, editorials, commentaries, abstracts only, trial registries, and letters to editors were excluded.

A comprehensive search strategy was developed with assistance from an information specialist (LR) (Appendix S1). We searched six databases from inception until March 2025. Specifically, we searched MEDLINE, Embase, Emcare and CENTRAL (Cochrane Central Register of Controlled Trials) from inception up to 18 March 2025, and Web of Science and CINAHL up to 19 March 2025. There was no limitation on language or year of publication. Google Translate was used for studies in a language other than English.

### 2.2 Study Selection

Endnote and Covidence software were used to manage citations [11]. Two reviewers (MM, AF, MC, SA or SB) independently screened titles/abstracts and full texts of identified studies. Any disagreements were resolved by discussion or consultation with a third reviewer (JPV). All RCTs identified for inclusion were assessed for research integrity and trustworthiness using published tools and guidance [12, 13, 14]. This ensured a transparent mechanism for identifying and managing problematic studies to avoid misleading findings.

### 2.3 Outcomes

The review outcomes were those used in previous WHO PPH recommendations and aligned with the core outcome set for treatment of PPH [15]. Primary outcomes were maternal death, additional blood loss ≥ 500 mL or ≥ 1000 mL, blood transfusion, additional uterotonics, other invasive nonsurgical interventions (including artery embolisation), surgical interventions (combined/unspecified e.g., hysterectomy, laparotomy, uterine artery ligation, internal iliac artery ligation, B‐Lynch suture, extensive vaginal repair), postpartum infections (e.g., sepsis).

Secondary outcomes were fever (maternal temperature ≥ 38°C), procedure‐related complications (e.g., trauma, necrosis), severe morbidity (e.g., vital organ failure or dysfunction, cardiac arrest, shock, intensive care unit admission), maternal hospital transfer, mean blood loss, postpartum anaemia (Hb < 9 g/dL), additional nonsurgical interventions (such as external aortic compression and compression garments), side effects (e.g., nausea, vomiting, shivering), delayed initiation of breastfeeding, prolonged hospitalisation, maternal well‐being, maternal satisfaction (pain levels and mobilisation), and breastfeeding at discharge.

### 2.4 Data Extraction

Data were extracted by two reviewers (MM, AF, MC, SA or SB) independently using a standardised extraction tool, with conflicts resolved by discussion or consultation with a third reviewer (JPV). When needed, we contacted authors of the included studies for additional information. Extracted data included author, year of publication, country, study design, population characteristics, sample size, definition of PPH, intervention and comparator characteristics and the outcomes of interest to this review.

### 2.5 Assessment of the Risk of Bias

The revised Cochrane risk‐of‐bias tool for randomised trials (RoB 2) was used to assess the quality of identified RCTs [16] and the ROBINS‐I tool for NRSIs [17]. Two reviewers independently assessed the risk of bias of the included studies, and disagreements were resolved through consensus. Studies with a critical risk of bias were excluded from data synthesis [17].

### 2.6 Data Analysis

Data from the RCT were not meta‐analysed but presented in a forest plot because only one RCT was included. For two outcomes of interest, only the median and inter‐quartile ranges were reported; therefore these were presented in box and whisker plots. Data from NRSI were analysed using DerSimonian and Laird random‐effects meta‐analyses in STATA SE 18 software [18]. Results were presented as risk ratios (RR) with 95% confidence intervals (CI), and heterogeneity was estimated via *T* ^2^ and *I* ^2^ statistics. The certainty of evidence was assessed using the Grading of Recommendations Assessment, Development and Evaluation (GRADE) tool [19].

The review was structured in such a way that we could capture studies across several possible comparisons. These comparisons are:

1. Comparison 1: Plain gauze versus balloon tamponade
2. Comparison 2: Gauze impregnated with haemostatic agents versus balloon tamponade
3. Comparison 3: Plain gauze versus uterine artery ligation or embolisation
4. Comparison 4: Gauze impregnated with haemostatic agents versus uterine artery ligation or embolisation

## 3 Results

The database search identified 2628 studies, with one additional study identified through citation tracking. After removing duplicates, 1874 titles/abstracts and 48 full texts were screened. Of these, 15 studies met the eligibility criteria: seven RCTs and eight NRSIs. Following research integrity assessment (RIA), six RCTs that did not pass assessment were classed as “awaiting classification”, pending authors' response to queries raised [20, 21, 22, 23, 24, 25]. Five RCTs were not registered [20, 21, 23, 24, 25], and one was retrospectively registered [22]. For the NRSIs, two studies were excluded, one due to editorial expression of concern [26] and the other due to critical risk of bias (Table S1) [27]. This resulted in one RCT [28] and six NRSIs contributing data for synthesis [29, 30, 31, 32, 33, 34] (Figure S1).

Three NRSIs were reported from the same hospital using the same intervention; data collection periods for all three studies overlapped (Table S2) [29, 30, 31]. The three studies reported multiple identical outcomes, though each also reported on a few unique outcomes. To avoid duplication, for each outcome, we used data only from the largest study providing data for that outcome.

The RCT included 204 women [28], and the NRSIs totalled 814 women, with sample sizes ranging from 75 [32] to 260 [30]. The three NRSIs with overlapping data collection periods had sample sizes of 78 [31], 136 [29] and 260 [30]. Studies were published from 2015 [33] to 2024 [30]. The RCT was conducted in China [28]; the three overlapping NRSIs were conducted in Germany [29, 30, 31]; two NRSIs were in China [32, 33]; and one NRSI in Pakistan [34]. The RCT recruited women with PPH following caesarean section (CS) for placenta previa (without suspected deeply invasive accreta) who had failed to respond to first‐line PPH treatment [28]. The NRSIs recruited women with PPH (≥ 500 mL blood loss after vaginal birth, or ≥ 1000 mL blood loss after CS) who did not respond to standard management with uterotonics, antifibrinolytic agents, volume replacement, uterine massage or manual compression. The causes of PPH in the NRSIs included uterine atony, obstetric trauma, retained placenta and placenta abnormalities (Table 1).

**TABLE 1:** Characteristics of studies included in the analysis.

Study	Country	Study population	Causes of postpartum haemorrhage	Study design	Data collection period	Sample size	Mode of birth (overall)	Intervention	Comparator
Wei 2020 [28]	China	Women with continuous bleeding after placental delivery following CS for placenta previa, who failed to respond to uterotonics, suturing and uterine devascularisation, and in the absence of suspected deeply invasive accreta	Placenta previa	RCT	Jun 2015 to Dec 2017	204	CS (*n* = 204)	Gauze packing (*n* = 102)	Double balloon catheter (*n* = 102)
Biele 2022 [29]	Germany	Women with PPH (≥ 500 mL blood loss after vaginal delivery or ≥ 1000 mL blood loss after CS) that did not respond to standard management (i.e., uterine massage, volume replacement, and standard medical treatment with uterotonic and antifibrinolytic agents)	Atony, obstetric injury, placental retention, placenta previa, placenta accreta spectrum disorder	Retrospective cohort	May 2016 to May 2019	136	Vaginal (*n* = 61) Instrumental^a^ (*n* = 14) CS (*n* = 61)	Chitosan^a^ gauze packing (*n* = 85)	Bakri balloon tamponade (*n* = 51)
Dueckelmann 2019 [31]	Germany	Women with PPH (≥ 500 mL blood loss after vaginal delivery or ≥ 1000 mL blood loss after CS) that did not respond to standard management (i.e., uterine massage, volume replacement, and standard medical treatment with uterotonic and antifibrinolytic agents)	Atony, obstetric injury, placental retention, placenta previa, abnormally invasive placenta	Retrospective cohort	Oct 2016 to Jun 2018	78	Vaginal (*n* = 34) Instrumental^a^ (*n* = 8) CS (*n* = 36)	Chitosan^a^ gauze packing (*n* = 47)	Bakri balloon tamponade (*n* = 31)
Dueckelmann 2024 [30]	Germany	Women with PPH (≥ 500 mL blood loss after vaginal delivery or ≥ 1000 mL blood loss after CS) that did not respond to standard management (i.e., uterine massage, volume replacement, and standard medical treatment with uterotonic and antifibrinolytic agents)	Atony, genital tract trauma, placenta previa, placenta accreta	Retrospective cohort	Jun 2016 to May 2021	260	Vaginal (*n* = 135) Instrumental^b^ (*n* = 33) CS (*n* = 92)	Chitosan^a^ gauze packing (*n* = 214)	Bakri balloon tamponade (*n* = 46)
Feng 2016 [32]	China	Women with post‐CS intractable PPH and ≥ 1500 mL blood loss within 1 h of delivery or bleeding leading to a coagulation disorder or multiple organ failure	Uterine inertia, placenta factor, genital injury, coagulation disorder	Retrospective cohort	May 2006 to May 2014	75	CS (*n* = 75)	Gauze packing (*n* = 33)	Uterine^c^ strapping (*n* = 25) or uterine artery embolisation (*n* = 17)
Guo 2015 [33]	China	Women with PPH who delivered via CS and used uterine gauze packing or balloon tamponade as second‐line treatment after failure of initial standard treatment (i.e., uterotonics, massage and manual compression)	Uterine inertia or placental factors	Retrospective cohort	Jan 2010 to Sept 2014	165	CS (*n* = 165)	Gauze packing (*n* = 99)	Balloon tamponade (*n* = 66)
Naeem 2022 [34]	Pakistan	Women with PPH admitted through an emergency with parity > 5 and failed to respond to medical treatment were included	Not clearly stated but women with PPH due to genital tract trauma, retained placenta or fibroids were excluded	Prospective cohort	Jan to Jun 2019	103	Vaginal (*n* = 62) Instrumental^a^ (*n* = 41)	Gauze packing (*n* = 53)	Balloon tamponade (*n* = 50)

^a^ Chitosan gauze is impregnated with a haemostatic agent, chitosan.

^b^ Instrumental delivery refers to the use of vacuum or forceps; CS, caesarean section.

^c^ Uterine strapping uses suture to strap the myometrium, thereby compressing and closing the sinusoids on the uterine placental separation surface; IV, intravenous; PPH, postpartum haemorrhage; RCT, randomised controlled trial.

The RCT and two NRSIs compared plain gauze to balloon tamponade (Comparison 1) [28, 33, 34]. The three NRSIs with overlapping data compared gauze impregnated with haemostatic agents to balloon tamponade (Comparison 2) [29, 30, 31]. Another NRSI compared plain gauze to artery ligation or embolisation (Comparison 3) [32]. No studies compared gauze impregnated with haemostatic agents to artery ligation or embolization (Comparison 4).

The RCT was judged to have ‘some concerns’ of bias in the selection of reported results due to the unavailability of a pre‐specified analysis plan (Figure S2). Two NRSIs were judged to have moderate risk of bias due to confounding [29, 33] and four were judged to have serious risk of bias due to confounding (Figure S3) [30, 31, 32, 34]. Reasons for downgrading the certainty of evidence included risk of bias, wide confidence interval, crossing the null and the appreciable thresholds for benefit and harm, small sample size (< 300 for dichotomous outcomes) and few events (Tables 2, 3, 4, 5, 6). The evidence from NRSIs was downgraded by two levels due to the inherent risk of bias associated with possible residual confounding and selection of participants (Tables 3, 4, 5, 6) [35].

**TABLE 2:** Summary of findings of all review outcomes from randomised controlled trials (*Comparison 1*).

Plain gauze compared to balloon tamponade for refractory postpartum haemorrhage					
Patient or population: refractory postpartum haemorrhage Intervention: Plain gauze Comparison: Balloon tamponade					
Outcomes	No. of participants (studies), follow‐up	Certainty of the evidence (GRADE)	Relative effect (95% CI)	Anticipated absolute effects	
				Risk with balloon tamponade	Risk difference with plain gauze
Additional surgical intervention	204 (1 RCT)	⨁⨁◯◯ Low^a^, ^b^	RR 1.29 (0.50–3.32)	69 per 1000	20 more per 1000 (34 fewer to 159 more)
Hysterectomy	204 (1 RCT)	⨁◯◯◯ Very low^a^, ^b^, ^c^	RR 3.00 (0.12–72.79)	0 per 1000	0 fewer per 1000 (0 fewer to 0 fewer)
Blood transfusion	204 (1 RCT)	⨁⨁◯◯ Low^a^, ^b^	RR 0.97 (0.67–1.41)	363 per 1000	11 fewer per 1000 (120 fewer to 149 more)
Postpartum anaemia < 11 g/dL	204 (1 RCT)	⨁⨁⨁◯ Moderate^b^	RR 1.27 (1.07–1.51)	647 per 1000	175 more per 1000 (45 more to 330 more)
ICU admissions	204 (1 RCT)	⨁◯◯◯ Very low^a^, ^b^, ^c^	RR 3.00 (0.12–72.79)	0 per 1000	0 fewer per 1000 (0 fewer to 0 fewer)
Infections	204 (1 RCT)	⨁◯◯◯ Very low^a^, ^b^, ^c^	RR 1.00 (0.06–15.77)	10 per 1000	0 fewer per 1000 (9 fewer to 145 more)
Fever	204 (1 RCT)	⨁⨁⨁◯ Moderate^b^	RR 2.40 (1.21–4.76)	98 per 1000	137 more per 1000 (21 more to 369 more)

*Note:* The risk in the intervention group (and its 95% confidence interval) is based on the assumed risk in the comparison group and the relative effect of the intervention (and its 95% CI). GRADE Working Group grades of evidence: High certainty: we are very confident that the true effect lies close to that of the estimate of the effect. Moderate certainty: we are moderately confident in the effect estimate: the true effect is likely to be close to the estimate of the effect, but there is a possibility that it is substantially different. Low certainty: our confidence in the effect estimate is limited: the true effect may be substantially different from the estimate of the effect. Very low certainty: we have very little confidence in the effect estimate: the true effect is likely to be substantially different from the estimate of effect.

Abbreviations: CI, confidence interval; RR, risk ratio.

^a^ Wide confidence interval including appreciable benefit and appreciable harm.

^b^ Estimate based on small sample size (< 300 for dichotomous outcomes).

^c^ Estimate based on a few events.

**TABLE 3:** Summary of findings of all review outcomes from non‐randomised studies of intervention (*Comparison 1*).

Plain gauze compared to balloon tamponade for refractory postpartum haemorrhage					
Patient or population: refractory postpartum haemorrhage Intervention: Plain gauze Comparison: Balloon tamponade					
Outcomes	No. of participants (studies), follow‐up	Certainty of the evidence (GRADE)	Relative effect (95% CI)	Anticipated absolute effects	
				Risk with balloon tamponade	Risk difference with plain gauze
Infection	103 (1 non‐randomised study)	⨁◯◯◯ Very low^a^, ^b^, ^c^, ^d^	RR 3.07 (1.07–8.78)	80 per 1000	166 more per 1000 (6 more to 622 more)

*Note:* The risk in the intervention group (and its 95% confidence interval) is based on the assumed risk in the comparison group and the relative effect of the intervention (and its 95% CI). GRADE Working Group grades of evidence: High certainty: we are very confident that the true effect lies close to that of the estimate of the effect. Moderate certainty: we are moderately confident in the effect estimate: the true effect is likely to be close to the estimate of the effect, but there is a possibility that it is substantially different. Low certainty: our confidence in the effect estimate is limited: the true effect may be substantially different from the estimate of the effect. Very low certainty: we have very little confidence in the effect estimate: the true effect is likely to be substantially different from the estimate of effect.

Abbreviations: CI, confidence interval; RR, risk ratio.

^a^ Study has a serious risk of bias due to confounding.

^b^ Inherent risk of bias associated with lack of randomisation.

^c^ Wide confidence interval.

^d^ Estimate based on small sample size (< 300 for dichotomous outcomes).

**TABLE 4:** Summary of findings of review outcomes from non‐randomised studies of interventions (*Comparison 2*).

Gauze impregnated with haemostatic agents compared to balloon tamponade for refractory postpartum haemorrhage					
Patient or population: refractory postpartum hemorrhage Intervention: Gauze impregnated with haemostatic agents Comparison: Balloon tamponade					
Outcomes	No. of participants (studies) Follow‐up	Certainty of the evidence (GRADE)	Relative effect (95% CI)	Anticipated absolute effects	
				Risk with balloon tamponade	Risk difference with gauze impregnated with haemostatic agents
Requiring additional intervention	136 (1 non‐randomised study)	⨁◯◯◯ Very low^a^, ^b^, ^c^	RR 0.60 (0.24–1.50)	157 per 1000	63 fewer per 1000 (119 fewer to 78 more)
Hysterectomy	136 (1 non‐randomised study)	⨁◯◯◯ Very low^a^, ^b^, ^c^, ^d^	RR 0.07 (0.00–1.22)	78 per 1000	73 fewer per 1000 (78 fewer to 17 more)
Laparotomy	136 (1 non‐randomised study)	⨁◯◯◯ Very low^a^, ^b^, ^c^, ^d^	RR 0.30 (0.06–1.58)	59 per 1000	41 fewer per 1000 (55 fewer to 34 more)
Blood transfusion	260 (1 non‐randomised study)	⨁◯◯◯ Very low^a^, ^b^, ^c^, ^e^	RR 0.98 (0.66–1.46)	391 per 1000	8 fewer per 1000 (133 fewer to 180 more)
ICU admission	260 (1 non‐randomised study)	⨁◯◯◯ Very low^a^, ^c^, ^e^	RR 0.55 (0.39–0.77)	543 per 1000	245 fewer per 1000 (332 fewer to 125 fewer)
Fever	260 (1 non‐randomised study)	⨁◯◯◯ Very low^a^, ^b^, ^c^, ^e^	RR 2.36 (0.31–17.86)	22 per 1000	30 more per 1000 (15 fewer to 367 more)
Severe morbidity	78 (1 non‐randomised study)	⨁◯◯◯ Very low^a^, ^b^, ^c^, ^d^, ^e^	RR 0.33 (0.03–3.48)	65 per 1000	43 fewer per 1000 (63 fewer to 160 more)
Prolonged hospitalisation > 3 days	246 (1 non‐randomised study)	⨁◯◯◯ Very low^a^, ^b^, ^c^, ^e^	RR 0.91 (0.71–1.17)	636 per 1000	57 fewer per 1000 (185 fewer to 108 more)
Maternal satisfaction	89 (1 non‐randomised study)	⨁◯◯◯ Very low^a^, ^b^, ^c^, ^e^	RR 1.07 (0.84–1.36)	800 per 1000	56 more per 1000 (128 fewer to 288 more)
Uterine artery embolisation	78 (1 non‐randomised study)	⨁◯◯◯ Very low^a^, ^b^, ^c^, ^e^	RR 2.00 (0.08–47.58)	0 per 1000	0 fewer per 1000 (0 fewer to 0 fewer)

*Note:* The risk in the intervention group (and its 95% confidence interval) is based on the assumed risk in the comparison group and the relative effect of the intervention (and its 95% CI). GRADE Working Group grades of evidence: High certainty: we are very confident that the true effect lies close to that of the estimate of the effect. Moderate certainty: we are moderately confident in the effect estimate: the true effect is likely to be close to the estimate of the effect, but there is a possibility that it is substantially different. Low certainty: our confidence in the effect estimate is limited: the true effect may be substantially different from the estimate of the effect. Very low certainty: we have very little confidence in the effect estimate: the true effect is likely to be substantially different from the estimate of effect.

Abbreviations: CI, confidence interval; RR, risk ratio.

^a^ Inherent risk of bias associated with lack of randomisation.

^b^ Wide confidence interval including appreciable benefit and appreciable harm.

^c^ Estimate based on small sample size (< 300 for dichotomous outcomes).

^d^ Estimate based on few events.

^e^ Study has a serious risk of bias due to confounding.

### 3.1 Findings From RCT (Comparison 1)

The median estimated blood loss was higher with gauze packing (312.6 mL, IQR: 223–450) compared to balloon tamponade (120 mL, IQR: 70–182.5) (Figure S4A). Uterine packing with plain gauze may not reduce the requirement for additional surgical intervention (RR 1.29, 95% CI 0.50–3.32, *low certainty evidence*) or blood transfusion (RR 0.97, 95% CI 0.67–1.41, *low certainty evidence*). Compared with balloon tamponade, plain gauze packing probably increases postpartum anaemia (< 11 g/dL) (relative risk (RR) 1.27, 95% CI 1.07–1.51) and maternal fever > 38°C (RR 2.40, 95% CI 1.21–4.76) (204 women; *moderate certainty evidence*) (Table 2). The evidence is very uncertain about the effect of plain gauze packing compared with balloon tamponade regarding the frequency of hysterectomy, ICU admission or infection (Figure S5; Table 2). The median postpartum pain score at 8 h using the visual analogue scale was similar between the two groups (Figure S4B).

### 3.2 Findings From NRSIs

The evidence from NRSIs is very uncertain about the effect of uterine packing with plain gauze or gauze impregnated with haemostatic agents compared to balloon tamponade (*Comparisons 1 and 2*) on any review outcome (*very low certainty evidence*) (Figures S6–S8, Tables 3, 4, 5). The evidence from NRSIs is also very uncertain about the effect of plain gauze packing compared to uterine artery ligation/embolisation (*Comparison 3*) on any review outcome (*very low certainty evidence*) (Figure S9, Table 6).

**TABLE 5:** Summary of findings of review outcomes from non‐randomised studies of interventions (*Comparisons 1 and 2*).

Plain gauze or gauze impregnated with haemostatic agent compared to balloon tamponade for refractory postpartum haemorrhage					
Patient or population: refractory postpartum haemorrhage Intervention: Gauze with or without haemostatic agent Comparison: Balloon tamponade.					
Outcomes	No. of participants (studies) follow‐up	Certainty of the evidence (GRADE)	Relative effect (95% CI)	Anticipated absolute effects	
				Risk with balloon tamponade	Risk difference with Gauze
Requiring additional intervention	404 (3 non‐randomised studies)	⨁◯◯◯ Very low^a^, ^b^, ^c^, ^d^	RR 0.88 (0.49–1.58)	108 per 1000	13 fewer per 1000 (55 fewer to 63 more)
Uterine artery embolisation	243 (2 non‐randomised studies)	⨁◯◯◯ Very low^b^, ^d^, ^e^, ^f^, ^g^	RR 0.92 (0.37–2.25)	72 per 1000	6 fewer per 1000 (45 fewer to 90 more)
Fever	425 (2 non‐randomised studies)	⨁◯◯◯ Very low^b^, ^d^, ^e^, ^f^	RR 0.88 (0.40–1.92)	89 per 1000	11 fewer per 1000 (54 fewer to 82 more)

*Note:* The risk in the intervention group (and its 95% confidence interval) is based on the assumed risk in the comparison group and the relative effect of the intervention (and its 95% CI). GRADE Working Group grades of evidence: High certainty: we are very confident that the true effect lies close to that of the estimate of the effect. Moderate certainty: we are moderately confident in the effect estimate: the true effect is likely to be close to the estimate of the effect, but there is a possibility that it is substantially different. Low certainty: our confidence in the effect estimate is limited: the true effect may be substantially different from the estimate of the effect. Very low certainty: we have very little confidence in the effect estimate: the true effect is likely to be substantially different from the estimate of effect.

Abbreviations: CI, confidence interval; RR, risk ratio.

^a^ One study with serious risk of bias, two with moderate risk of bias.

^b^ Inherent risk of bias due to lack of randomisation.

^c^ Moderate heterogeneity *I* ^2^ = 54.63%; 1 of 3 studies favours balloon tamponade.

^d^ Wide confidence interval including appreciable benefit and appreciable harm.

^e^ One of two studies with serious risk of bias.

^f^ Point estimates favour different arms.

^g^ Estimate based on small sample size (< 300 for dichotomous outcomes).

**TABLE 6:** Summary of findings of all review outcomes from non‐randomised studies of intervention (*Comparison 3*).

Plain gauze compared to uterine artery ligation or embolisation for refractory postpartum haemorrhage					
Patient or population: refractory postpartum haemorrhage Intervention: Plain gauze Comparison: uterine artery ligation or embolisation					
Outcomes	No. of participants (studies) Follow‐up	Certainty of the evidence (GRADE)	Relative effect (95% CI)	Anticipated absolute effects	
				Risk with ligation or embolisation	Risk difference with plain gauze
Hysterectomy	75 (1 non‐randomised study)	⨁◯◯◯ Very low^a^, ^b^, ^c^, ^d^	RR 0.85 (0.26–2.76)	143 per 1000	21 fewer per 1000 (106 fewer to 251 more)
Maternal death	75 (1 non‐randomised study)	⨁◯◯◯ Very low^a^, ^b^, ^c^, ^d^, ^e^	RR 3.79 (0.16–90.22)	0 per 1000	0 fewer per 1000 (0 fewer to 0 fewer)
Haemostatic success	75 (1 non‐randomised study)	⨁◯◯◯ Very low^a^, ^b^, ^c^, ^d^	RR 1.00 (0.79–1.27)	786 per 1000	0 fewer per 1000 (165 fewer to 212 more)

*Note:* The risk in the intervention group (and its 95% confidence interval) is based on the assumed risk in the comparison group and the relative effect of the intervention (and its 95% CI). GRADE Working Group grades of evidence: High certainty: we are very confident that the true effect lies close to that of the estimate of the effect. Moderate certainty: we are moderately confident in the effect estimate: the true effect is likely to be close to the estimate of the effect, but there is a possibility that it is substantially different. Low certainty: our confidence in the effect estimate is limited: the true effect may be substantially different from the estimate of the effect. Very low certainty: we have very little confidence in the effect estimate: the true effect is likely to be substantially different from the estimate of effect.

Abbreviations: CI, confidence interval; RR, risk ratio.

^a^ Study has serious risk of bias due to confounding.

^b^ Inherent risk of bias due to lack of randomisation.

^c^ Wide confidence interval including appreciable benefit and appreciable harm.

^d^ Estimate based on small sample size (< 300 for dichotomous outcomes).

^e^ Estimate based on few events.

## 4 Discussion

### 4.1 Main Findings

We analysed data from one RCT and six NRSIs on the effectiveness and safety of uterine packing with plain gauze or gauze impregnated with haemostatic agent(s). Although we initially identified 15 eligible studies, six RCTs and one NRSI were excluded because of integrity concerns, and one NRSI was excluded due to critical risk of bias. Estimated blood loss was higher after gauze packing than after balloon tamponade in the included RCT. Low‐certainty evidence from the RCT suggests that compared to balloon tamponade, plain gauze packing might make no difference in the need for additional surgical intervention or blood transfusion. Moderate certainty evidence from the RCT showed that, compared to balloon tamponade, plain gauze packing probably increases postpartum anaemia and maternal fever. From NRSIs, the evidence is very uncertain about the effect of uterine packing with plain gauze or gauze impregnated with haemostatic agents on any review outcome.

### 4.2 Strengths and Limitations

Strengths of this review include adherence to rigorous Cochrane systematic review methods and PRISMA guidance, making it a robust and up‐to‐date synthesis on the safety and effectiveness of uterine packing with plain gauze or gauze impregnated with haemostatic agents for treating refractory PPH. We assessed all RCTs and contacted authors for a comprehensive assessment of research integrity, to ensure this review was devoid of problematic or potentially fraudulent data. Trial authors were asked to provide clarification on the quality issues we identified, though none responded.

Our review has some limitations—due to limited evidence from high‐quality RCTs, we considered it necessary to consider NRSIs [36]. Although we only included NRSIs with an appropriate comparator, these study designs have a higher risk of selection bias, particularly the five included NRSIs that used retrospective designs. Also, three of the NRSIs included data from the same hospital across overlapping time periods [29, 30, 31]. For these studies, we only included the largest study with available data per review outcome to avoid duplication. Furthermore, due to the very low certainty of evidence from NRSIs, we are uncertain of the effects of uterine gauze packing on refractory PPH from these studies.

### 4.3 Interpretation

To our knowledge, there are no prior systematic reviews examining the safety and effectiveness of uterine packing with plain gauze or gauze impregnated with haemostatic agents specifically for refractory PPH. However, two prior reviews have included studies with uterine gauze packing as a comparator [9, 37]. A Cochrane review by Kellie et al. [9] compared the safety and effectiveness of mechanical and surgical interventions used for the treatment of primary PPH. The review considered only RCTs, and identified one where uterine gauze packing was the comparator for the uterine balloon tamponade intervention [21]. That 2018 RCT by Ashraf et al. [21] is “awaiting classification” within our review, due to the lack of trial registration. Consistent with our findings, Kellie et al. [9] concluded that there was insufficient evidence to determine whether uterine packing is safe or effective. Another systematic review and meta‐analysis in 2023 by Abul et al. [37] examined the safety and efficacy of intrauterine balloon tamponade compared with uterine gauze packing for treating primary PPH. They concluded that intrauterine balloon tamponade was a safer and more effective treatment option for PPH than uterine gauze packing [37]. Neither of these prior reviews used trial trustworthiness assessments [12, 14].

The available evidence does not indicate any benefits of uterine gauze packing for refractory PPH. Evidence from one RCT—where postpartum anaemia and fever were greater in women randomised to plain gauze packing rather than balloon tamponade—signals it may possibly cause harm. The RCT included women with placenta previa diagnosed before birth [28]. There is also insufficient evidence to ascertain whether impregnating gauze with haemostatic agents makes any difference. Only three non‐randomised studies (with overlapping data from the same hospital) used gauze impregnated with a haemostatic agent, chitosan [29, 30, 31]. Chitosan promotes platelet activation and the agglutination of blood proteins to facilitate fibrin clot formation [38, 39]. However, the certainty of available evidence is very low, and we cannot draw conclusions on the benefits or harms of this approach. We therefore agree with those clinical guidelines that recommend against uterine gauze packing for treating refractory PPH outside of a research protocol [4, 40].

High‐quality RCTs are needed to provide definitive answers on the effectiveness and safety of this intervention. The lack of sufficient RCTs is somewhat unsurprising—refractory PPH is an emergency and relatively infrequent, meaning such trials are operationally challenging. Nevertheless, seven RCTs of this intervention have been published [20, 21, 22, 23, 24, 25, 28]. Future trials of uterine packing will likely need to recruit in multiple centres and large hospitals where refractory PPH is more common. Different approaches to informed consent—such as seeking (pre)consent prior to or at hospital admission, in the event a PPH might occur—would allow women to be randomised efficiently [41].

## 5 Conclusion

The evidence regarding uterine packing with plain gauze or gauze impregnated with haemostatic agents for treating refractory PPH is limited—we analysed data from one small RCT and several NRSIs only. The review found no evidence of benefit and there is the potential for harm when plain gauze is used for refractory PPH in women with placenta praevia undergoing caesarean section. There is no evidence of benefit or harm in other clinical scenarios which is concerning. Prospective, well‐controlled RCTs are needed to evaluate this intervention's benefits and harms and ascertain whether it has any role to play in the PPH treatment pathway. There is currently insufficient evidence to recommend uterine gauze packing with plain gauze or gauze impregnated with haemostatic agents for the treatment of refractory PPH.

## Author Contributions

M.M. and J.P.V. drafted the protocol. M.M. and L.R. developed the search strategy. M.M., A.F., M.C., S.B., S.A. selected the studies, extracted data and conducted quality assessment. M.M. and J.P.V. conducted the data analysis. M.M. wrote the first draft of the manuscript. All authors contributed to the interpretation of the findings and critically revised successive drafts of the manuscript.

## Funding

This study was funded by UNDP/UNFPA/UNICEF/WHO/World Bank Special Programme of Research, Development and Research Training in Human Reproduction (HRP), a co‐sponsored programme executed by the World Health Organization. Sarah Boudova was supported by the Society for Maternal‐Fetal Medicine Queenan Global Health Award International Agency Mentored Research Fellowship. The views expressed in this manuscript represent those of the named authors and not necessarily that of the funders (HRP).

## Conflicts of Interest

The authors declare no conflicts of interest.

## Supporting information


**Appendix S1:** Search strategy.

**Table S1:** Characteristics of studies awaiting classification or excluded from the analysis.
**Table S2:** Studies contributing data by outcome for the three overlapping studies.
**Figure S1:** PRISMA flow chart of included studies.
**Figure S2:** Risk of bias of randomised controlled trial.
**Figure S3:** Risk of bias of non‐randomised studies of intervention.
**Figure S4:** Box and whisker plot of (A) estimated blood loss after gauze packing or balloon tamponade (B) postpartum pain score (Wei 2020).
**Figure S5:** Forest plots of review outcomes from the randomised controlled trial, Wei 2020.
**Figure S6:** Forest plots of review outcomes from NRSIs comparing plain gauze versus balloon tamponade (Comparison 1 only).
**Figure S7:** Forest plots of review outcomes from NRSIs comparing gauze impregnated with haemostatic agents versus balloon tamponade (Comparison 2 only). (A) hysterectomy (B) laparotomy (C) blood transfusion (D) ICU admission (E) severe maternal morbidity (F) prolonged hospitalisation > 3 days (G) maternal satisfaction.
**Figure S8:** Forest plots of review outcomes from NRSIs by subgroups with plain gauze or gauze impregnated with haemostatic agent versus balloon tamponade (Comparisons 1 and 2). (A) Additional surgical/radiological intervention (B) uterine artery embolization (C) fever.
**Figure S9:** Forest plots of review outcomes from NRSIs comparing plain gauze uterine artery ligation or embolization (Comparison 3 only).

## Data Availability

The data that supports the findings of this study is available within the article and its Supporting Information.

## References

[1] J. L. Bienstock , A. C. Eke , and N. A. Hueppchen , “Postpartum Hemorrhage,” New England Journal of Medicine 384, no. 17 (2021): 1635–1645. 33913640 10.1056/NEJMra1513247 PMC10181876

[2] J. A. Cresswell , M. Alexander , M. Y. C. Chong , et al., “Global and Regional Causes of Maternal Deaths 2009–20: A WHO Systematic Analysis,” Lancet Global Health 13 (2025): e626–e634. 40064189 10.1016/S2214-109X(24)00560-6 PMC11946934

[3] World Health Organization , “A Roadmap to Combat Postpartum Haemorrhage Between 2023 and 2030,” (World Health Organization, 2023).

[4] “WHO Recommendations for the Prevention and Treatment of Postpartum Haemorrhage,” (Geneva: World Health Organization, 2012). 23586122

[5] I. Gallos , A. Devall , J. Martin , et al., “Randomized Trial of Early Detection and Treatment of Postpartum Hemorrhage,” New England Journal of Medicine 389, no. 1 (2023): 11–21. 37158447 10.1056/NEJMoa2303966

[6] “WHO Recommendation on Tranexamic Acid for the Treatment of Postpartum Haemorrhage,” (2017). 29630190

[7] W. R. Parry Smith , A. Papadopoulou , E. Thomas , et al., “Uterotonic Agents for First‐Line Treatment of Postpartum Haemorrhage: A Network Meta‐Analysis,” Cochrane Database of Systematic Reviews 11, no. 11 (2020): Cd012754. 33232518 10.1002/14651858.CD012754.pub2 PMC8130992

[8] M. Widmer , G. Piaggio , G. J. Hofmeyr , et al., “Maternal Characteristics and Causes Associated With Refractory Postpartum Haemorrhage After Vaginal Birth: A Secondary Analysis of the WHO CHAMPION Trial Data,” BJOG: An International Journal of Obstetrics and Gynaecology 127, no. 5 (2020): 628–634. 31808245 10.1111/1471-0528.16040 PMC7078998

[9] F. J. Kellie , J. N. Wandabwa , H. A. Mousa , and A. D. Weeks , “Mechanical and Surgical Interventions for Treating Primary Postpartum Haemorrhage,” Cochrane Database of Systematic Reviews 7 (2020): CD013663. 32609374 10.1002/14651858.CD013663 PMC8407481

[10] J. P. T. Higgins , J. Thomas , J. Chandler , M. Cumpston , T. Li , and M. J. Page , “Cochrane Handbook for Systematic Reviews of Interventions Version 6.2 (updated February 2021),” (2021).

[11] “Covidence Systematic Review Software, Veritas Health Innovation, Melbourne, Australia,” July 7, 2023, www.covidence.org.

[12] S. Weibel , M. Popp , S. Reis , N. Skoetz , P. Garner , and E. Sydenham , “Identifying and Managing Problematic Trials: A Research Integrity Assessment (RIA) Tool for Randomized Controlled Trials in Evidence Synthesis,” medRxiv (2022). 10.1002/jrsm.1599 PMC10551123 36054583

[13] Z. Alfirevic , F. J. Kellie , J. Weeks , et al., “Identifying and Handling Potentially Untrustworthy Trials,” Trustworthiness Screening Tool (TST) (2023).

[14] Trustworthiness Criteria for Meta‐Analyses of Randomized Controlled Studies: OBGYN Journal Guidelines,” BJOG: An International Journal of Obstetrics and Gynaecology 132, no. 1 (2025): 1–5. 39356178 10.1111/1471-0528.17945 PMC11612606

[15] S. Meher , A. Cuthbert , J. J. Kirkham , et al., “Core Outcome Sets for Prevention and Treatment of Postpartum Haemorrhage: An International Delphi Consensus Study,” BJOG: An International Journal of Obstetrics and Gynaecology 126, no. 1 (2019): 83–93. 29920912 10.1111/1471-0528.15335

[16] J. A. C. Sterne , J. Savović , M. J. Page , et al., “RoB 2: A Revised Tool for Assessing Risk of Bias in Randomised Trials,” BMJ (Clinical Research Ed.) 366 (2019): l4898. 10.1136/bmj.l4898 31462531

[17] J. A. Sterne , M. A. Hernán , A. McAleenan , B. C. Reeves , and J. P. Higgins , “Assessing Risk of Bias in a Non‐Randomized Study,” in Cochrane Handbook for Systematic Reviews of Interventions (Cochrane, 2019), 621–641.

[18] StataCorp , “Stata Statistical Software: Release 18,” (College Station, TX: StataCorp LLC, 2023).

[19] “GRADEpro GDT: GRADEpro Guideline Development Tool [Software],” McMaster University and Evidence Prime (2022), www.gradepro.org/.

[20] S. Agrawal and S. I. Munir , “Comparison of Efficacy and Safety of Intrauterine Balloon Tamponade Versus Uterovaginal Packing in Females Presenting With Postpartum Hemorrhage After Normal Vaginal Delivery,” Nepal Medical College Journal 26, no. 1 (2024): 34–38.

[21] N. Ashraf , A. Ashraf , and K. Khursheed , “Efficacy and Safety of Intrauterine Balloon Tamponadeversus Uterovaginal Roll Gauze Packing in Patient Presenting With Primary Postpartum Hemorrhage After Normal Vaginal Delivery,” Annals of King Edward Medical University 24, no. S (2018): 889–892.

[22] Y. Dai , J. Wei , Z. Wang , et al., “Intrauterine Balloon Tamponade Combined With Temporary Abdominal Aortic Balloon Occlusion in the Management of Women With Placenta Accreta Spectrum: A Randomized Controlled Trial,” Zhonghua Fu Chan Ke Za Zhi 55, no. 7 (2020): 450–456. 32842248 10.3760/cma.j.cn112141-20200225-00135

[23] S. U. Nisa , S. U. Nisa , M. Athar , N. Zulfiqar , B. A. Malik , and N. Leghari , “Comparison Between Balloon Inflation and Uterovaginal Packing After Vaginal Delivery in Term of Control of Primary PPH,” Pakistan Journal of Medical & Health Sciences 17, no. 04 (2023): 153–155.

[24] F. Rehman and P. Noor ul Amina , “Comparison of Efficacy (Control of Primary Post‐Partum Hemorrhage) After Vaginal Delivery Between Balloon Inflation and Uterovaginal Packing,” Pakistan Journal of Medical & Health Sciences 16, no. 06 (2022): 242–243.

[25] S. Ujala , N. Shaheen , R. Khicchi , and A. Masood , “Comparison of Efficacy of Balloon Inflation and Uterovaginal Packing for Control of Primary Postpartum Hemorrhage After Vaginal Delivery,” Rawal Medical Journal 46, no. 4 (2021): 877–879.

[26] E. Elshamy , M. Rezk , and A.‐E. Shaheen , “Is It Worth to Insert Uterine Pack Instead of Bakri Balloon to Control Postpartum Hemorrhage After Vaginal Delivery in Hypertensive Patients?,” Archives of Gynecology and Obstetrics 307, no. 4 (2023): 1195–1201. 35396973 10.1007/s00404-022-06543-y

[27] X. Yue , X. Zhang , A. Liu , S. Cui , and P. Yang , “Clinical Analysis of 223 Cases of Postpartum Hemorrhage,” Journal of Zhengzhou University: Medical Edition 40, no. 2 (2005): 361–363.

[28] J. Wei , Y. Dai , Z. Wang , et al., “Intrauterine Double‐Balloon Tamponade vs Gauze Packing in the Management of Placenta Previa: A Multicentre Randomized Controlled Trial,” Medicine 99, no. 7 (2020): e19221. 32049861 10.1097/MD.0000000000019221 PMC7035072

[29] C. Biele , L. Radtke , L. Kaufner , et al., “Does the Use of Chitosan Covered Gauze for Postpartum Hemorrhage Reduce the Need for Surgical Therapy Including Hysterectomy? A Databased Historical Cohort Study,” Journal of Perinatal Medicine 50, no. 8 (2022): 1078–1086. 35611816 10.1515/jpm-2021-0533

[30] A. M. Dueckelmann , P. Hermann , C. Biele , et al., “Short and Long‐Term Menstrual, Reproductive, and Mental Health Outcomes After the Intrauterine Use of Chitosan Tamponade or the Bakri Balloon for Severe Postpartum Hemorrhage: An Observational Study,” Journal of Maternal‐Fetal & Neonatal Medicine 37, no. 1 (2024): 2354382. 38782738 10.1080/14767058.2024.2354382

[31] A. M. Dueckelmann , L. Hinkson , A. Nonnenmacher , et al., “Uterine Packing With Chitosan‐Covered Gauze Compared to Balloon Tamponade for Managing Postpartum Hemorrhage,” European Journal of Obstetrics, Gynecology, and Reproductive Biology 240 (2019): 151–155. 31284089 10.1016/j.ejogrb.2019.06.003

[32] B. Feng , J. Zhai , and Y. Cai , “Results of Surgical Intervention in Treating Post‐Cesarean Intractable Postpartum Hemorrhage,” International Journal of Clinical and Experimental Medicine 9, no. 2 (2016): 5154–5160.

[33] Y.‐N. Guo , J. Ma , X.‐J. Wang , and B.‐S. Wang , “Does Uterine Gauze Packing Increase the Risk of Puerperal Morbidity in the Management of Postpartum Hemorrhage During Caesarean Section: A Retrospective Cohort Study,” International Journal of Clinical and Experimental Medicineis 8, no. 8 (2015): 13740–13747. PMC4613005 26550320

[34] S. Naeem , F. P. Memon , H. Najam , and A. Memon , “Intrauterine Balloon Tamponade Versus Gauze Packing for Treatment of Post‐Partum Hemorrhage,” Journal of Liaquat University of Medical & Health Sciences 21, no. 03 (2022): 185–189.

[35] H. J. Schünemann , J. P. Higgins , G. E. Vist , et al., “Completing ‘Summary of Findings’ Tables and Grading the Certainty of the Evidence,” in Cochrane Handbook for Systematic Reviews of Interventions (Cochrane, 2019), 375–402.

[36] B. C. Reeves , J. J. Deeks , J. P. Higgins , et al., “Including Non‐Randomized Studies on Intervention Effects,” in Cochrane Handbook for Systematic Reviews of Interventions (John Wiley & Sons, 2019), 595–620.

[37] A. Abul , A. Al‐Naseem , A. Althuwaini , A. Al‐Muhanna , and N. S. Clement , “Safety and Efficacy of Intrauterine Balloon Tamponade vs Uterine Gauze Packing in Managing Postpartum Hemorrhage: A Systematic Review and Meta‐Analysis,” AJOG Global Reports 3, no. 1 (2023): 100135. 36578464 10.1016/j.xagr.2022.100135 PMC9791175

[38] D. Gheorghiță , H. Moldovan , A. Robu , et al., “Chitosan‐Based Biomaterials for Hemostatic Applications: A Review of Recent Advances,” International Journal of Molecular Sciences 24, no. 13 (2023): 10540. 37445718 10.3390/ijms241310540 PMC10342007

[39] M. S. Lord , B. Cheng , S. J. McCarthy , M. Jung , and J. M. Whitelock , “The Modulation of Platelet Adhesion and Activation by Chitosan Through Plasma and Extracellular Matrix Proteins,” Biomaterials 32, no. 28 (2011): 6655–6662. 21676458 10.1016/j.biomaterials.2011.05.062

[40] M. F. Escobar , A. H. Nassar , G. Theron , et al., “FIGO Recommendations on the Management of Postpartum Hemorrhage 2022,” International Journal of Gynaecology and Obstetrics 157, no. Suppl 1 (2022): 3–50. 35297039 10.1002/ijgo.14116 PMC9313855

[41] M. Widmer , M. Bonet , and A. P. Betrán , “Would You Like to Participate in This Trial? The Practice of Informed Consent in Intrapartum Research in the Last 30 Years,” PLoS One 15, no. 1 (2020): e0228063. 31978100 10.1371/journal.pone.0228063 PMC6980544

