# Supplementary material for: Safety and Effectiveness of Uterine Gauze Packing for Refractory Postpartum Haemorrhage: A Systematic Review and Meta‐Analysis
Source: BJOG. 2025 Nov 21;133(4):579–89. doi: 10.1111/1471-0528.70091 (PMC12884202; doi:10.1111/1471-0528.70091)
Supplement: Supplementary file 1 — Appendix S1: Search strategy. [file BJO-133-579-s002.docx]

**Search 18 March 2025**

Database(s): **Ovid MEDLINE(R)**1946 to March 14, 2025
Search Strategy:

| **#** | **Searches** |
| --- | --- |
| 1 | (h?emostatic agent* or antih?emorrhagic* or anti-h?emor*agic*).mp. |
| 2 | Hemostatics/ |
| 3 | (chitosan adj3 (dressing* or cloth? or bandage or fabric? or pad? or padding)).mp. |
| 4 | (impregnated adj2 gauze).mp. |
| 5 | (impregnated adj2 h?emostatic*).mp. |
| 6 | (h?emostatic* adj2 (dressing* or cloth? or bandage or fabric? or pad? or padding)).mp. |
| 7 | (impregnated adj2 chitosan).mp. |
| 8 | (uterine packing or vaginal packing or uterovaginal packing or utero-vaginal packing).mp. |
| 9 | (intrauterine packing or intra-uterine packing).mp. |
| 10 | gauze.mp. |
| 11 | (kaolin adj3 (dressing* or cloth? or bandage or fabric? or pad? or padding)).mp. |
| 12 | (oxidi?ed cellulose adj3 (dressing* or cloth? or bandage or fabric? or pad? or padding)).mp. |
| 13 | Fibrin/ |
| 14 | 1 or 2 or 3 or 4 or 5 or 6 or 7 or 8 or 9 or 10 or 11 or 12 or 13 |
| 15 | Uterine Inertia/ |
| 16 | (uterine atony or atonic uterus or uterus atonia).mp. |
| 17 | Postpartum Hemorrhage/ |
| 18 | ((postpartum or post-partum or obstetric) adj (h?emor*age or bleeding or blood loss)).mp. |
| 19 | ((post-birth or post-delivery) adj h?emor*age).mp. |
| 20 | Uterine Inversion/ |
| 21 | (uterine inversion* or uterus inversion* or inverted uterus).mp. |
| 22 | Placenta, Retained/ |
| 23 | (placental retention or retained placenta*).mp. |
| 24 | Placenta Previa/ |
| 25 | (placenta praevia or placenta previa*).mp. |
| 26 | Placenta Accreta/ |
| 27 | (placenta* accreta or placental accretion).mp. |
| 28 | Postpartum Period/ |
| 29 | ((postpartum or post-partum) adj (wom?n or female?)).mp. |
| 30 | 15 or 16 or 17 or 18 or 19 or 20 or 21 or 22 or 23 or 24 or 25 or 26 or 27 or 28 or 29 |
| 31 | 14 and 30 |
| 32 | exp animals/ not humans.sh. |
| 33 | 31 not 32 |

Database(s): **Embase Classic+Embase**1947 to 2025 March 14
Search Strategy:

| **#** | **Searches** |
| --- | --- |
| 1 | (h?emostatic agent* or antih?emorrhagic* or anti-h?emor*agic*).mp. |
| 2 | Hemostatics/ |
| 3 | (chitosan adj3 (dressing* or cloth? or bandage or fabric? or pad? or padding)).mp. |
| 4 | hemostatic agent/ |
| 5 | (impregnated adj2 gauze).mp. |
| 6 | (impregnated adj2 h?emostatic*).mp. |
| 7 | (h?emostatic* adj2 (dressing* or cloth? or bandage or fabric? or pad? or padding)).mp. |
| 8 | (impregnated adj2 chitosan).mp. |
| 9 | (uterine packing or vaginal packing or uterovaginal packing or utero-vaginal packing).mp. |
| 10 | (intrauterine packing or intra-uterine packing).mp. |
| 11 | gauze.mp. |
| 12 | (kaolin adj3 (dressing* or cloth? or bandage or fabric? or pad? or padding)).mp. |
| 13 | (oxidi?ed cellulose adj3 (dressing* or cloth? or bandage or fabric? or pad? or padding)).mp. |
| 14 | Fibrin/ |
| 15 | 1 or 2 or 3 or 4 or 5 or 6 or 7 or 8 or 9 or 10 or 11 or 12 or 13 or 14 |
| 16 | (uterine atony or atonic uterus or uterine atonia).mp. |
| 17 | uterine atony/ |
| 18 | Uterine Inertia/ |
| 19 | uterus inversion/ |
| 20 | Postpartum Hemorrhage/ |
| 21 | ((postpartum or post-partum or obstetric) adj (h?emor*age or bleeding or blood loss)).mp. |
| 22 | ((post-birth or post-delivery) adj h?emor*age).mp. |
| 23 | retained placenta/ |
| 24 | (placental retention or retained placenta*).mp. |
| 25 | (placenta praevia or placenta previa*).mp. |
| 26 | placenta accreta/ |
| 27 | (placenta* accreta or placental accretion).mp. |
| 28 | puerperium/ |
| 29 | ((postpartum or post-partum) adj (wom?n or female?)).mp. |
| 30 | (uterine inversion* or uterus inversion* or inverted uterus).mp. |
| 31 | Placenta Previa/ |
| 32 | 17 or 18 or 19 or 20 or 21 or 22 or 23 or 24 or 25 or 26 or 27 or 28 or 29 or 30 or 31 |
| 33 | 15 and 32 |
| 34 | (exp animal/ or nonhuman/ or animal.hw. or invertebrates/) not human.sh. |
| 35 | 33 not 34 |

Database(s): **Ovid Emcare**1995 to 2025 Week 10
Search Strategy:

| **#** | **Searches** |
| --- | --- |
| 1 | (h?emostatic agent* or antih?emorrhagic* or anti-h?emor*agic*).mp. |
| 2 | Hemostatics/ |
| 3 | (chitosan adj3 (dressing* or cloth? or bandage or fabric? or pad? or padding)).mp. |
| 4 | hemostatic agent/ |
| 5 | (impregnated adj2 gauze).mp. |
| 6 | (impregnated adj2 h?emostatic*).mp. |
| 7 | (h?emostatic* adj2 (dressing* or cloth? or bandage or fabric? or pad? or padding)).mp. |
| 8 | (impregnated adj2 chitosan).mp. |
| 9 | (uterine packing or vaginal packing or uterovaginal packing or utero-vaginal packing).mp. |
| 10 | (intrauterine packing or intra-uterine packing).mp. |
| 11 | gauze.mp. |
| 12 | (kaolin adj3 (dressing* or cloth? or bandage or fabric? or pad? or padding)).mp. |
| 13 | (oxidi?ed cellulose adj3 (dressing* or cloth? or bandage or fabric? or pad? or padding)).mp. |
| 14 | Fibrin/ |
| 15 | 1 or 2 or 3 or 4 or 5 or 6 or 7 or 8 or 9 or 10 or 11 or 12 or 13 or 14 |
| 16 | (uterine atony or atonic uterus or uterine atonia).mp. |
| 17 | uterine atony/ |
| 18 | Uterine Inertia/ |
| 19 | uterus inversion/ |
| 20 | Postpartum Hemorrhage/ |
| 21 | ((postpartum or post-partum or obstetric) adj (h?emor*age or bleeding or blood loss)).mp. |
| 22 | ((post-birth or post-delivery) adj h?emor*age).mp. |
| 23 | retained placenta/ |
| 24 | (placental retention or retained placenta*).mp. |
| 25 | (placenta praevia or placenta previa*).mp. |
| 26 | placenta accreta/ |
| 27 | (placenta* accreta or placental accretion).mp. |
| 28 | puerperium/ |
| 29 | ((postpartum or post-partum) adj (wom?n or female?)).mp. |
| 30 | (uterine inversion* or uterus inversion* or inverted uterus).mp. |
| 31 | Placenta Previa/ |
| 32 | 17 or 18 or 19 or 20 or 21 or 22 or 23 or 24 or 25 or 26 or 27 or 28 or 29 or 30 or 31 |
| 33 | 15 and 32 |
| 34 | (exp animal/ or nonhuman/ or animal.hw. or invertebrates/) not human.sh. |
| 35 | 33 not 34 |

Database(s): **EBM Reviews - Cochrane Central Register of Controlled Trials**February 2025
Search Strategy:

| **#** | **Searches** |
| --- | --- |
| 1 | (h?emostatic agent* or antih?emorrhagic* or anti-h?emor*agic*).mp. |
| 2 | Hemostatics/ |
| 3 | (chitosan adj3 (dressing* or cloth? or bandage or fabric? or pad? or padding)).mp. |
| 4 | (impregnated adj2 (gauze or h?emostatic* or chitosan)).mp. |
| 5 | ((h?emostatic* or kaolin or oxidi?ed cellulose) adj3 (dressing* or cloth? or bandage or fabric? or pad? or padding)).mp. |
| 6 | (uterine packing or vaginal packing or uterovaginal packing or utero-vaginal packing).mp. |
| 7 | (intrauterine packing or intra-uterine packing).mp. |
| 8 | gauze.mp. |
| 9 | Fibrin/ |
| 10 | 1 or 2 or 3 or 4 or 5 or 6 or 7 or 8 or 9 |
| 11 | (uterine atony or atonic uterus or uterine atonia).mp. |
| 12 | uterine atony/ |
| 13 | Uterine Inertia/ |
| 14 | Postpartum Hemorrhage/ |
| 15 | ((postpartum or post-partum or obstetric) adj (h?emor*age or bleeding or blood loss)).mp. |
| 16 | Placenta, Retained/ |
| 17 | (placental retention or retained placenta*).mp. |
| 18 | (placenta praevia or placenta previa*).mp. |
| 19 | placenta accreta/ |
| 20 | Abruptio Placentae/ |
| 21 | Placenta Previa/ |
| 22 | (placenta* accreta or placental accretion).mp. |
| 23 | puerperium/ |
| 24 | ((postpartum or post-partum) adj (wom?n or female?)).mp. |
| 25 | (uterine inversion* or uterus inversion* or inverted uterus).mp. |
| 26 | 11 or 12 or 13 or 14 or 15 or 16 or 17 or 18 or 19 or 20 or 21 or 22 or 23 or 24 or 25 |
| 27 | 10 and 26 |

**Database: CINAHL (Date of search 19^th^ March 2025)**

| **#** | **Query** | **Limiters/Expanders** | **Last Run Via** |
| --- | --- | --- | --- |
| S33 | S31 AND S32 | Search modes - Find all my search terms | Interface - EBSCOhost Research Databases Search Screen - Advanced Search Database - CINAHL Complete |
| S32 | S14 OR S15 OR S16 OR S17 OR S18 OR S19 OR S20 OR S21 OR S22 OR S23 OR S24 OR S25 OR S29 OR S30 | Search modes - Find any of my search terms | Interface - EBSCOhost Research Databases Search Screen - Advanced Search Database - CINAHL Complete |
| S31 | S1 OR S2 OR S3 OR S4 OR S5 OR S6 OR S7 OR S8 OR S9 OR S10 OR S11 OR S12 OR S13 OR S26 OR S27 OR S28 | Search modes - Find any of my search terms | Interface - EBSCOhost Research Databases Search Screen - Advanced Search Database - CINAHL Complete |
| S30 | MH "Postpartum Hemorrhage" | Search modes - Find any of my search terms | Interface - EBSCOhost Research Databases Search Screen - Advanced Search Database - CINAHL Complete |
| S29 | MH "Uterine Inertia" | Search modes - Find any of my search terms | Interface - EBSCOhost Research Databases Search Screen - Advanced Search Database - CINAHL Complete |
| S28 | MH "Fibrin" | Search modes - Find any of my search terms | Interface - EBSCOhost Research Databases Search Screen - Advanced Search Database - CINAHL Complete |
| S27 | “h?emostatic agent*” or antih?emor*agic* or “anti-h?emor*agic*” | Search modes - Find any of my search terms | Interface - EBSCOhost Research Databases Search Screen - Advanced Search Database - CINAHL Complete |
| S26 | MH "Hemostatics" | Search modes - Find any of my search terms | Interface - EBSCOhost Research Databases Search Screen - Advanced Search Database - CINAHL Complete |
| S25 | "postpartum wom?n" | Search modes - Find any of my search terms | Interface - EBSCOhost Research Databases Search Screen - Advanced Search Database - CINAHL Complete |
| S24 | postpartum | Search modes - Find all my search terms | Interface - EBSCOhost Research Databases Search Screen - Advanced Search Database - CINAHL Complete |
| S23 | "placenta accreta" | Search modes - Find all my search terms | Interface - EBSCOhost Research Databases Search Screen - Advanced Search Database - CINAHL Complete |
| S22 | "placenta previa" | Search modes - Find all my search terms | Interface - EBSCOhost Research Databases Search Screen - Advanced Search Database - CINAHL Complete |
| S21 | "placenta* abruption" or "abruptio placent#e" | Search modes - Find all my search terms | Interface - EBSCOhost Research Databases Search Screen - Advanced Search Database - CINAHL Complete |
| S20 | "retained placenta" | Search modes - Find all my search terms | Interface - EBSCOhost Research Databases Search Screen - Advanced Search Database - CINAHL Complete |
| S19 | "uterine inversion*" or "uterus inversion*" or "inverted uterus" | Search modes - Find all my search terms | Interface - EBSCOhost Research Databases Search Screen - Advanced Search Database - CINAHL Complete |
| S18 | "Uterine Inversion" | Search modes - Find all my search terms | Interface - EBSCOhost Research Databases Search Screen - Advanced Search Database - CINAHL Complete |
| S17 | ((postpartum or “post-partum” or obstetric) N0 (h?emor*age or bleeding or “blood loss”)) | Search modes - Find all my search terms | Interface - EBSCOhost Research Databases Search Screen - Advanced Search Database - CINAHL Complete |
| S16 | "Postpartum Hemorrhage" | Search modes - Find all my search terms | Interface - EBSCOhost Research Databases Search Screen - Advanced Search Database - CINAHL Complete |
| S15 | "uterine atony" or "atonic uterus" or "uterus atonia" | Search modes - Find all my search terms | Interface - EBSCOhost Research Databases Search Screen - Advanced Search Database - CINAHL Complete |
| S14 | "Uterine Inertia" | Search modes - Find all my search terms | Interface - EBSCOhost Research Databases Search Screen - Advanced Search Database - CINAHL Complete |
| S13 | fibrin | Search modes - Find all my search terms | Interface - EBSCOhost Research Databases Search Screen - Advanced Search Database - CINAHL Complete |
| S12 | ("oxidi?ed cellulose") N2 (dressing* or cloth* or bandage or fabric* or pad* or padding) | Search modes - Proximity | Interface - EBSCOhost Research Databases Search Screen - Advanced Search Database - CINAHL Complete |
| S11 | kaolin N2 (dressing* or cloth* or bandage or fabric* or pad* or padding) | Search modes - Proximity | Interface - EBSCOhost Research Databases Search Screen - Advanced Search Database - CINAHL Complete |
| S10 | gauze | Search modes - Find all my search terms | Interface - EBSCOhost Research Databases Search Screen - Advanced Search Database - CINAHL Complete |
| S9 | "intrauterine packing" or "intra-uterine packing" | Search modes - Find all my search terms | Interface - EBSCOhost Research Databases Search Screen - Advanced Search Database - CINAHL Complete |
| S8 | "uterine packing" or "vaginal packing" or "uterovaginal packing" or "utero-vaginal packing" | Search modes - Find all my search terms | Interface - EBSCOhost Research Databases Search Screen - Advanced Search Database - CINAHL Complete |
| S7 | impregnated N1 chitosan | Search modes - Proximity | Interface - EBSCOhost Research Databases Search Screen - Advanced Search Database - CINAHL Complete |
| S6 | h?emostatic N2 (dressing* or cloth* or bandage or fabric* or pad* or padding) | Search modes - Proximity | Interface - EBSCOhost Research Databases Search Screen - Advanced Search Database - CINAHL Complete |
| S5 | impregnated N2 h#emostatic* | Search modes - Proximity | Interface - EBSCOhost Research Databases Search Screen - Advanced Search Database - CINAHL Complete |
| S4 | impregnated N1 gauze | Search modes - Proximity | Interface - EBSCOhost Research Databases Search Screen - Advanced Search Database - CINAHL Complete |
| S3 | chitosan N2 (dressing* or cloth* or bandage or fabric* or pad* or padding) | Search modes - Proximity | Interface - EBSCOhost Research Databases Search Screen - Advanced Search Database - CINAHL Complete |
| S2 | antih#emorrhagic | Search modes - Find any of my search terms | Interface - EBSCOhost Research Databases Search Screen - Advanced Search Database - CINAHL Complete |
| S1 | "h#emostatic agents" | Search modes - Find any of my search terms | Interface - EBSCOhost Research Databases Search Screen - Advanced Search Database - CINAHL Complete |

**Web of Science Search Strategy (v0.1)**

Date Run: Wed Mar 19 2025

# Database: All Databases

# Entitlements:

- WOS: 1900 to 2025

- BIOABS: 1980 to 2025

- BIOSIS: 1926 to 2025

- CSCD: 1989 to 2025

- CCC: 1998 to 2025

- DRCI: 1900 to 2025

- GRANTS: 1953 to 2025

- KJD: 1980 to 2025

- MEDLINE: 1950 to 2025

- PPRN: 1991 to 2025

- PQDT: 1637 to 2025

- SCIELO: 2002 to 2025

# Searches:

1: (TS=(“h?emostatic agent*” or antih?emor*agic* or “anti-h?emor*agic*”))

2: (TS=((chitosan or kaolin or “oxidi?ed cellulose”) NEAR/2 (dressing* or cloth? or bandage or

fabric? or pad? or padding)))

3: TS=(“impregnated gauze”)

4: TS=(“impregnated h?emostatic agent*”)

5: TS=(chitosan)

6: TS=(kaolin)

7: TS=(“oxidi?ed cellulose”)

8: (TS=(“uterine packing” or “vaginal packing” or “uterovaginal packing” or “intrauterine

Packing”))

9: (TS=(gauze))

10: TS=(fibrin)

11: #10 OR #9 OR #8 OR #7 OR #6 OR #5 OR #4 OR #3 OR #2 OR #1

12: TS=(uterine inertia)

13: TS=(uterine atony)

14: TS=(uterine atony)

15: TS=(uterine atony)

16: TS=(“uterine atony”) and “Uterine Atony” (OR – Search within topic) and “Postpartum

H?emor*age” (OR – Search within topic) and Atony (OR – Search within topic) and “Atonía

Uterina” (OR – Search within topic)

17: (TS=(uterine inversion))

18: TS=(“uterus inversion” or “inverted uterus”)

19: TS=(“retained placenta” or “placental retention”)

20: TS=(“retained placenta” or “placental retention”) and “Retained Placenta” (OR – Search within topic) and “Placental Retention” (OR – Search within topic)

21: TS=(“placenta pr#evia” or “placenta* accret*” or “placenta abruptio*”)

22: TS=(“postpartum wom?n” or “postpartum period”)

23: TS=(((postpartum or “post-partum” or obstetric) NEAR/0 (h?emor*age or bleeding or “blood loss”)))

24: #23 OR #22 OR #21 OR #20 OR #19 OR #18 OR #17 OR #16 OR #15 OR #14 OR #13 OR #12

25: #24 AND #11

26: #25 and Review Article or Case Report or Letter or Meeting or Awarded Grant

or Biography or Retraction or Expression Of Concern or Retracted Publication or Dissertation

Thesis or Data Set or Correction (Exclude – Document Types) and Editorial Material (Document

Types) and Abstract (Document Types)
