# Supplementary material for: Safety and Effectiveness of Uterine Gauze Packing for Refractory Postpartum Haemorrhage: A Systematic Review and Meta‐Analysis
Source: BJOG. 2025 Nov 21;133(4):579–89. doi: 10.1111/1471-0528.70091 (PMC12884202; doi:10.1111/1471-0528.70091)
Supplement: Supplementary file 2 — Table S1: Characteristics of studies awaiting classification or excluded from the analysis. Table S2: Studies contributing data by outcome for the three overlapping studies. Figure S1: PRISMA flow chart of included studies. Figure S2: Risk of bias of randomised controlled trial. Figure S3: Risk of bias of non‐randomised studies of intervention. Figure S4: Box and whisker plot of (A) estimated blood loss after gauze packing or balloon tamponade (B) postpartum pain score (Wei 2020). Figure S5: Forest plots of review outcomes from the randomised controlled trial, Wei 2020. Figure S6: Forest plots of review outcomes from NRSIs comparing plain gauze versus balloon tamponade (Comparison 1 only). Figure S7: Forest plots of review outcomes from NRSIs comparing gauze impregnated with haemostatic agents versus balloon tamponade (Comparison 2 only). (A) hysterectomy (B) laparotomy (C) blood transfusion (D) ICU admission (E) severe maternal morbidity (F) prolonged hospitalisation > 3 days (G) maternal satisfaction. Figure S8: Forest plots of review outcomes from NRSIs by subgroups with plain gauze or gauze impregnated with haemostatic agent versus balloon tamponade (Comparisons 1 and 2). (A) Additional surgical/radiological intervention (B) uterine artery embolization (C) fever. Figure S9: Forest plots of review outcomes from NRSIs comparing plain gauze uterine artery ligation or embolization (Comparison 3 only). [file BJO-133-579-s001.docx]

# Supplementary Tables and Figures: Safety and effectiveness of uterine gauze packing for refractory postpartum haemorrhage: A systematic review and meta-analysis

**Table S1. Characteristics of studies awaiting classification or excluded from the analysis**

| **Study** | **Country** | **Population** | **Study design** | **Sample size** | **Intervention** | **Comparator** | **Reason for exclusion** |
| --- | --- | --- | --- | --- | --- | --- | --- |
| Agrawal 2024^1^ | Pakistan | Women with primary PPH after vaginal delivery at term (> 37 weeks) unresponsive to medical treatment. Women with PPH due to perineal, cervical or vaginal tear, episiotomy, retained placenta, normal vaginal delivery after one previous cesarean section, coagulation disorder, secondary PPH were excluded | RCT | 212 | Gauze packing (n = 106) | Balloon tamponade  (n = 106) | Failed trustworthiness assessment |
| Ashraf 2018^2^ | Pakistan | Women with primary PPH (≥500ml blood loss with 24hrs) after vaginal delivery at term (> 37 weeks) unresponsive to medical treatment. Women with PPH due to perineal, cervical or vaginal tear, episiotomy, retained placenta, normal vaginal delivery after one previous cesarean section, coagulation disorder, secondary PPH were excluded | RCT | 212 | Gauze packing (n = 106) | Balloon tamponade  (n = 106) | Failed trustworthiness assessment |
| Dai 2020^3^ | China | Women with previous CS, placenta adherence syndrome, planned CS and uterus retention. | RCT | 161 | Gauze packing (n = 80) | Hemostatic bag (n = 81) | Failed trustworthiness assessment |
| Nisa 2023^4^ | Pakistan | Women with PPH after vaginal delivery. Women with twin pregnancy, a history of CS or a bleeding disorder were excluded. | RCT | 140 | Gauze packing (n = 70) | Balloon inflation (n = 70) | Failed trustworthiness assessment |
| Rehman 2022^5^ | Pakistan | Women with PPH after vaginal delivery. Women with twin pregnancy, a history of CS or a bleeding disorder were excluded. | RCT | 120 | Gauze packing (n = 60) | Balloon inflation (n = 60) | Failed trustworthiness assessment |
| Ujala 2021^6^ | Pakistan | Women with PPH after vaginal delivery. Women with twin pregnancy, a history of CS or a bleeding disorder were excluded. | RCT | 104 | Gauze packing (n = 52) | Balloon inflation (n = 52) | Failed trustworthiness assessment |
| Elshamy 2023^7^ | Saudi Arabia | Women with atonic PPH (estimated blood loss ≥500 ml) after routine administration of 5 IU oxytocin by slow IV infusion; who were not responding to further first-line measures (bimanual compression of the uterus and administration of uterotonic agents in the form of 10 IU oxytocin by IV infusion) | Prospective cohort | 142 | Gauze packing  (n = 68)  (gauze soaked in povidone-iodine solution) | Bakri balloon  (n = 74) | Editorial expression of concern |
| Yue 2005^8^ | China | Women with PPH | Retrospective cohort | 223 | Uterine tamponade (n = 64) | Conservative treatment (n = 159) | Critical risk of bias |

CS, caesarean section; IV, intravenous; PPH, postpartum haemorrhage; RCT, randomised controlled trial

Table S2. Studies contributing data by outcome for the three overlapping studies

| Outcomes | Biele 2022^9^  May 2016 to May 2019 | Dueckelmann 2024^10^  Jun 2016 to May 2021 | Dueckelmann 2019^11^  Oct 2016 to Jun 2018 |
| --- | --- | --- | --- |
| Additional surgical intervention |  |  |  |
| Hysterectomy |  |  |  |
| Laparotomy |  |  |  |
| Blood transfusion |  |  |  |
| ICU admission |  |  |  |
| Fever |  |  |  |
| Severe maternal morbidity |  |  |  |
| Prolonged hospitalization >3days |  |  |  |
| Maternal satisfaction |  |  |  |

Green indicates that the study contributed data to the outcome, while red indicates the study did not contribute data. Only one study contributed to each outcome.


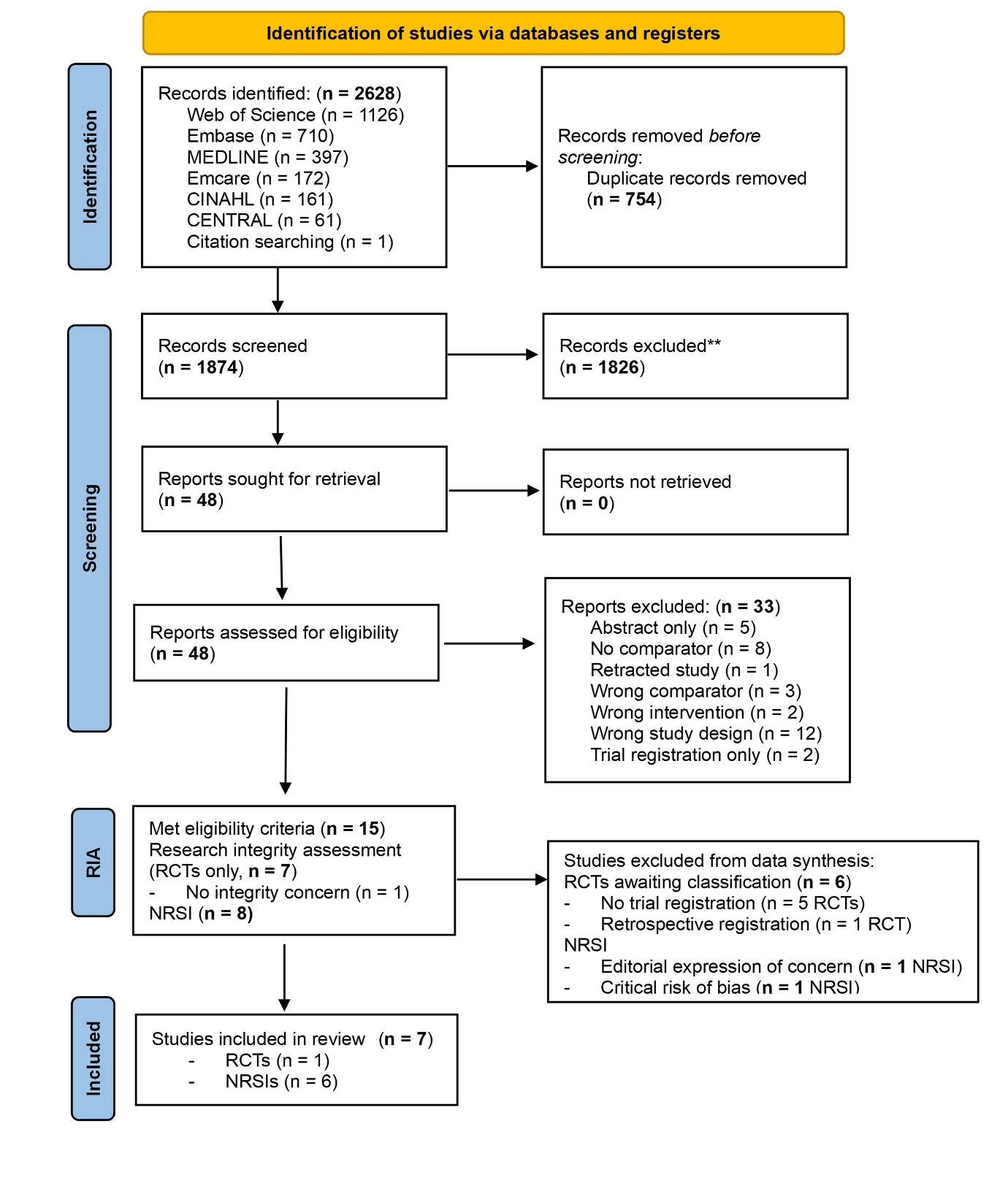


**Figure S1. PRISMA flow chart of included studies**

RCTs (Randomised Controlled Trials); NRSIs (Non-Randomised Studies of Intervention); RIA (Research Integrity Assessment)


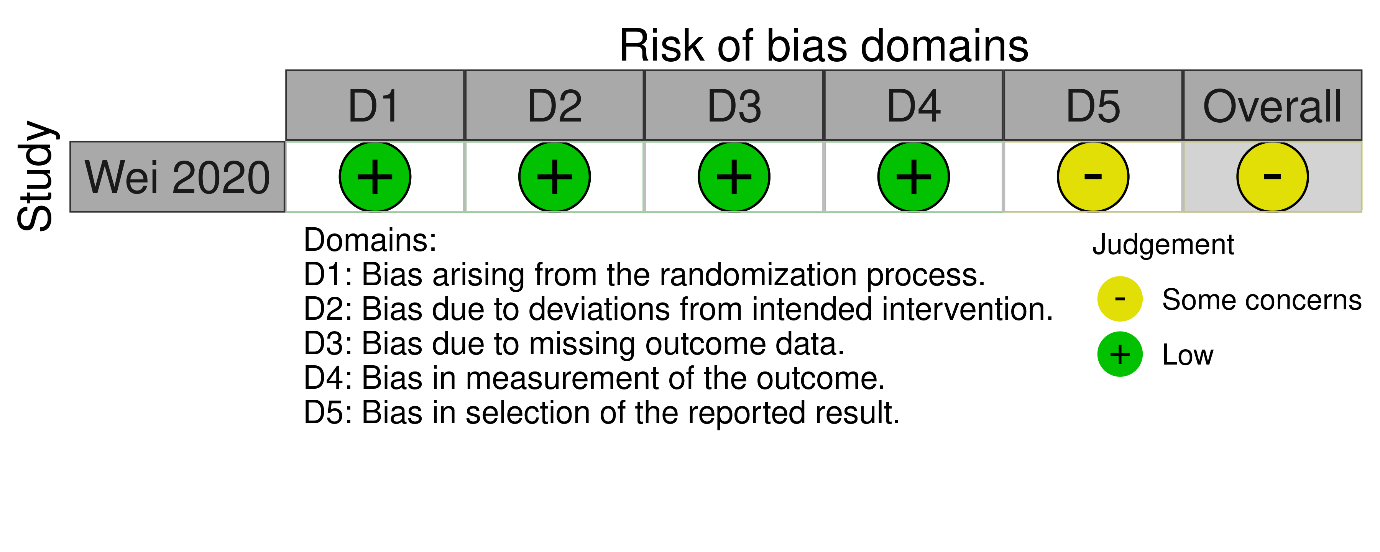

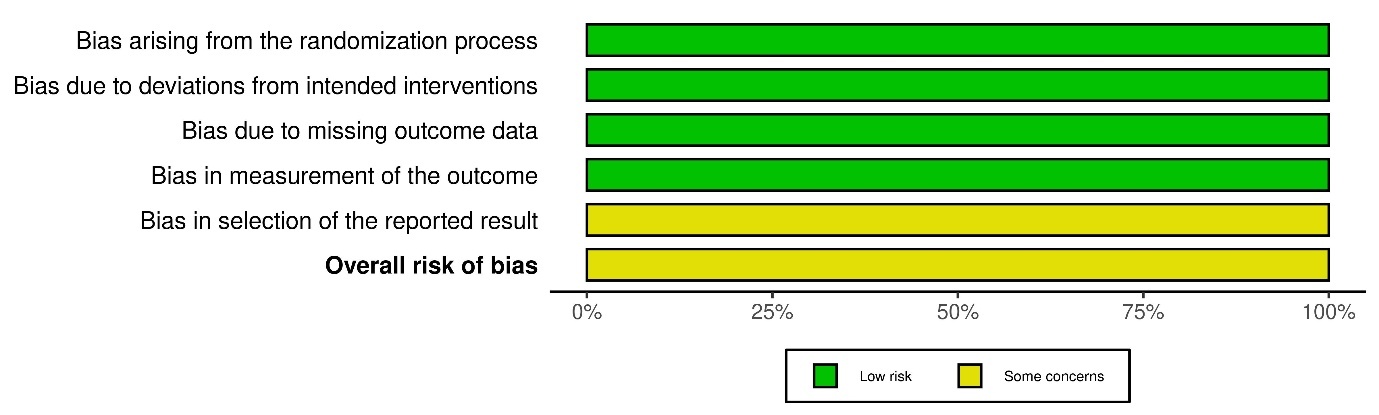


**Figure S2. Risk of bias of randomised controlled trial**


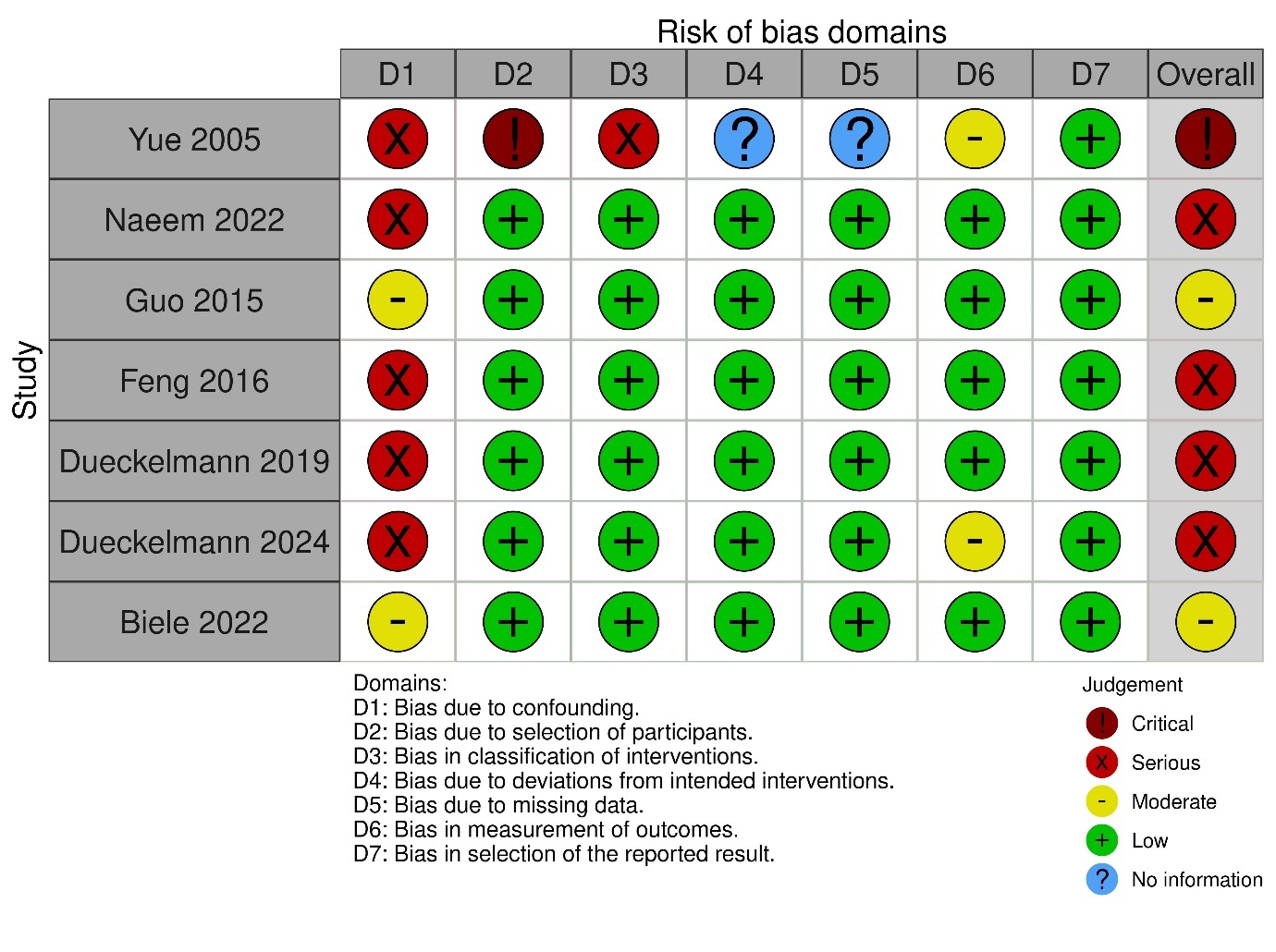


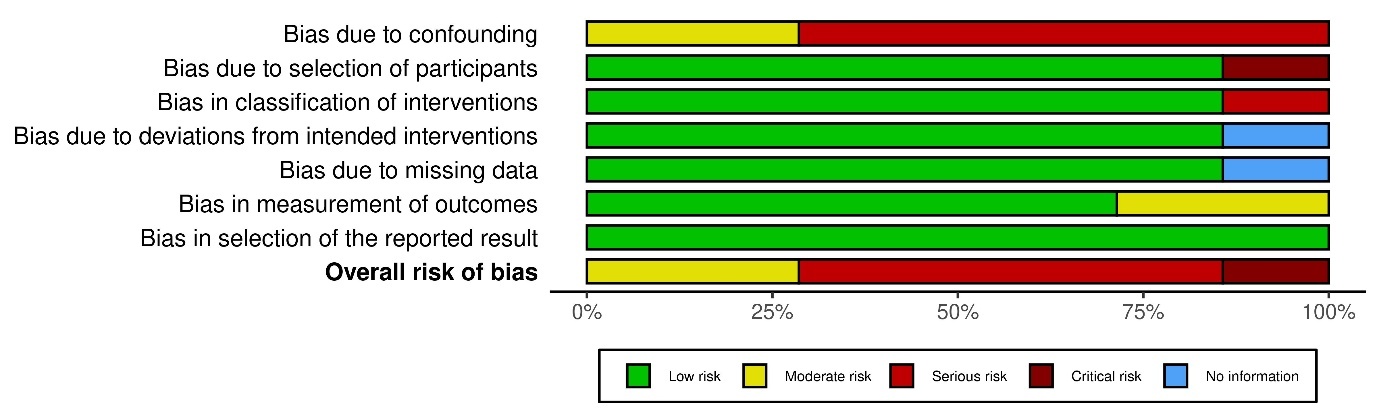


**Figure S3. Risk of bias of non-randomised studies of intervention**

**A B**

**Figure S4. Box and whisker plot of (A) estimated blood loss after gauze packing or balloon tamponade (B) postpartum pain score (Wei 2020)**

Postpartum pain score measured at 8 hours using the visual analogue scale

Figure S5. Forest plots of review outcomes from the randomised controlled trial, Wei 2020

Comparison 1: Plain gauze versus balloon tamponade

Additional surgical/radiological intervention refers to the number of women requiring additional surgical or radiological interventions (uterine artery embolisation, hysterectomy or replacement with another form of intrauterine tamponade) to achieve haemostasis; Blood transfusion refers to the number of women requiring allogeneic blood transfusion; Postpartum anaemia was measured as haemoglobin <11g/dl; All women were given prophylactic antibiotic (cefazolin 1 g) every 8 hours until the gauze packing or balloon was removed; Fever of >38^o^C on any 2 of the first 10 days (measured 4 times each day with an interval of more than 4 hours) following delivery exclusive of the first 24 hours.

**A**

Figure S6. Forest plots of review outcomes from NRSIs comparing plain gauze versus balloon tamponade (Comparison 1 only)

**A**

**B**

**C**

**D**

**E**

**F**

**G**

Figure S7. Forest plots of review outcomes from NRSIs comparing gauze impregnated with haemostatic agents versus balloon tamponade (Comparison 2 only)

A) hysterectomy B) laparotomy C) blood transfusion D) ICU admission E) severe maternal morbidity F) prolonged hospitalisation >3days G) maternal satisfaction

NRSIs in this comparison: Biele 2022 (May 2016 - May 2019); Dueckelmann 2019 (June 2016 - June 2018); Dueckelmann 2024 (October 2016 - May 2021) Data overlap from these studies, so only the largest was used where outcomes were reported

(Figure S7C) refers to the number of women who received blood transfusion

(Figure S7E) severe morbidity included kidney failure after extensive blood loss and consequently cardiomyopathy; resuscitation needed due to cardiac decompensation because of amniotic fluid embolism after emergency c-section

(Figure S7G) refers to maternal satisfaction with the treatment

**A**

**B**

**C**

Figure S8. Forest plots of review outcomes from NRSIs by subgroups with plain gauze or gauze impregnated with haemostatic agent versus balloon tamponade (Comparisons 1 and 2)

A) Additional surgical/radiological intervention B) uterine artery embolization C) fever

(NRSIs in this comparison: Biele 2022^a^, Dueckelmann 2019^a^, Dueckelmann 2024^a^, Guo 2015, Naeem 2022)

^a^Because of data overlap in these studies, only the largest one with outcome data was included per analysis

(Figure S8A) Defined as the number of women requiring additional surgical or radiological treatment (hysterectomy, laparotomy or embolisation) after gauze packing or balloon tamponade to achieve haemostasis

(Figure S8C) Fever >38^o^C

**A**

**B**

**C**

**Figure S9. Forest plots of review outcomes from NRSIs comparing plain gauze uterine artery ligation or embolization (Comparison 3 only)**

NRSI in the comparison: Feng 2016

Haemostatic success was defined as: firm uterine contractions; vaginal bleeding ≤50 mL/h, gradually reducing or stopping; stable vital signs; normal urine volume

**References**

1. Agrawal S, Munir SI. Comparison of Efficacy and Safety of Intrauterine Balloon Tamponade Versus Uterovaginal Packing in Females Presenting with Postpartum Hemorrhage after Normal Vaginal Delivery. Nepal Medical College Journal. 2024;26(1):34-8.

2. Ashraf N, Ashraf A, Khursheed K. Efficacy and Safety of Intrauterine Balloon Tamponadeversus Uterovaginal Roll Gauze Packing in Patient Presenting with Primary Postpartum Hemorrhage after Normal Vaginal Delivery. Annals of King Edward Medical University. 2018;24(S):889-92.

3. Dai Y, Wei J, Wang Z, Zhang X, Cheng L, Gu N, et al. Intrauterine balloon tamponade combined with temporary abdominal aortic balloon occlusion in the management of women with placenta accreta spectrum: a randomized controlled trial. Zhonghua fu Chan ke za zhi. 2020;55(7):450-6.

4. Nisa SU, Nisa SU, Athar M, Zulfiqar N, Malik BA, Leghari N. Comparison between Balloon Inflation and Uterovaginal packing after Vaginal Delivery in Term of Control of Primary PPH. Pakistan Journal of Medical & Health Sciences. 2023;17(04):153-5.

5. Rehman F, Noor ul Amina P. Comparison of Efficacy (Control of Primary Post-Partum Hemorrhage) after Vaginal Delivery between Balloon Inflation and Uterovaginal Packing. Pakistan Journal of Medical & Health Sciences. 2022;16(06):242-3.

6. Ujala S, Shaheen N, Khicchi R, Masood A. Comparison of efficacy of balloon inflation and uterovaginal packing for control of primary postpartum hemorrhage after vaginal delivery. Rawal Medical Journal. 2021;46(4):877-9.

7. Elshamy E, Rezk M, Shaheen A-E. Is it worth to insert uterine pack instead of Bakri balloon to control postpartum hemorrhage after vaginal delivery in hypertensive patients? Archives of Gynecology and Obstetrics. 2023;307(4):1195-201.

8. Yue X, Zhang X, Liu A, Cui S, Yang P. Clinical analysis of 223 cases of postpartum hemorrhage. Journal of Zhengzhou University: Medical Edition. 2005;40(2):361-3.

9. Biele C, Radtke L, Kaufner L, Hinkson L, Braun T, Henrich W, et al. Does the use of chitosan covered gauze for postpartum hemorrhage reduce the need for surgical therapy including hysterectomy? A databased historical cohort study. Journal of Perinatal Medicine. 2022;50(8):1078-86.

10. Dueckelmann AM, Hermann P, Biele C, Leichtle C, Waldner C, Braun T, et al. Short and long-term menstrual, reproductive, and mental health outcomes after the intrauterine use of chitosan tamponade or the Bakri balloon for severe postpartum hemorrhage: an observational study. The Journal of Maternal-Fetal & Neonatal Medicine. 2024;37(1):2354382.

11. Dueckelmann AM, Hinkson L, Nonnenmacher A, Siedentopf J-P, Schoenborn I, Weizsaecker K, et al. Uterine packing with chitosan-covered gauze compared to balloon tamponade for managing postpartum hemorrhage. European Journal of Obstetrics & Gynecology and Reproductive Biology. 2019;240:151-5.
